# Effect of post-ICU follow-up by a rapid response team after congenital heart surgery

**DOI:** 10.1038/s41598-022-09683-y

**Published:** 2022-04-04

**Authors:** Taiki Haga, Takaaki Sakaguchi, Takao Kazuta, Takaya Morooka, Junji Maruyama, Naoko Yamaoka, Satoko Miyahara

**Affiliations:** 1 Department of Paediatric Critical Care Medicine, Osaka City General Hospital, 2-13-22, Miyakojima-hondori, Miyakojima-ku, Osaka, Osaka 534-0021 Japan; 2 Department of Emergency and Critical Care Medical Centre, Osaka City General Hospital, 2-13-22, Miyakojima-hondori, Miyakojima-ku, Osaka, Osaka 534-0021 Japan; 3 Nursing Department, Intensive Care Centre, Osaka City General Hospital, 2-13-22, Miyakojima-hondori, Miyakojima-ku, Osaka, Osaka 534-0021 Japan; 4 Department of Medical Security and Patient Safety, Osaka City General Hospital, 2-13-22, Miyakojima-hondori, Miyakojima-ku, Osaka, Osaka 534-0021 Japan

**Keywords:** Physiology, Cardiovascular biology

## Abstract

Patients with congenital heart disease who have a variety of cardiac/extracardiac problems are at high risk for deterioration. This study aimed to determine the effectiveness of post-intensive care unit (ICU) follow-up by a rapid response team (RRT) after congenital heart surgery. This before-and-after study was conducted at an urban regional tertiary hospital. We enrolled 572 consecutive patients who underwent congenital heart surgery and were transferred alive from the paediatric ICU (PICU) between April 2015 and March 2020. Post-ICU follow-up for 48 h was started in April 2018. The primary and secondary endpoints were unplanned ICU readmission and clinical outcomes at ICU readmission, respectively. Overall, 346 and 226 patients were analysed pre- and post-intervention, respectively. Patient demographics were similar between groups, but in the post-intervention group, patients tended to have had more complicated surgery. Unplanned ICU readmission rates within 30 days were similar between groups. Regarding the demographics and outcomes at ICU readmission, patients in the post-intervention group had lower predicted mortality rates (1.7% vs 5.3%, *P* = 0.001), required less ventilator days (median, 0.5 days [interquartile range (IQR) 0–1] vs median, 3 days [IQR 0.5–4], *P* = 0.02), and had a shorter ICU stay (median, 3 days [IQR 2–4] vs median, 6 days [IQR 3–9], *P* = 0.03), but there was no significant between-group difference in ICU mortality. Post-ICU follow-up by a RRT after congenital heart surgery did not decrease unplanned ICU readmission but improved several outcomes at ICU readmission.

## Introduction

Congenital heart disease (CHD) presents with a wide range of physiologic/anatomic circulatory abnormalities. The majority of patients with CHD continue to have various cardiac/extracardiac problems that need ongoing care and management^1^. Given their vulnerability, they have higher morbidity and mortality risks than the general population^2, 3^ and can become seriously ill from only the slightest perturbations, such as infection or injury.

The rapid response system (RRS) is one strategy to improve the prognosis of patients with sudden deterioration in the hospital. The RRS is a medical safety system that detects early signs of most serious conditions, such as respiratory and/or cardiac arrest, and prevents such situations. A team of specially trained professionals, such as a medical emergency team and rapid response team (RRT), needs to be organized to immediately respond to the needs of patients when their condition worsens. Various factors can trigger the RRS, such as abnormalities in the patient’s vital signs, concerns of medical personnel/patient’s family, or the follow-up of high-risk patients^4^. Recently, a systematic review of adult patients has reported that follow-ups by a specialized team after intensive care unit (ICU) transfer reduced unplanned ICU readmissions^5^. Meanwhile, it has been reported that deteriorating patients with CHD who required unscheduled ICU readmission had a poor outcome^6, 7^. Taken together, we hypothesize that post-ICU transfer follow-ups for patients with CHD would decrease unplanned ICU readmissions and improve their outcomes, but there are no previous reports to show this.

Therefore, we conducted a single-centre before-and-after study to examine whether post-ICU follow-ups by a specialized RRT reduce unscheduled ICU readmissions in patients after CHD surgery and reduce the severity of the illness and the prognosis of patients who require readmission.

## Methods

### Study design and setting

A before-and-after study was conducted at an urban regional tertiary hospital in Osaka, Japan; the 3-year before-implementation period (April 2015 to March 2018) was compared to the 2-year after-implementation period (April 2018 to March 2020) (here, we refer to the implementation of post-ICU follow-up by the RRT). This hospital has a multidisciplinary paediatric intensive care unit (PICU) with 12 beds; all patients, regardless of age, were admitted to the PICU after CHD surgery. Approximately 100 to 150 congenital heart surgeries are performed in this hospital annually. The protocol for this study was approved by the ethics committee of Osaka City General Hospital (reference number: 1805021) as being in compliance with the 1975 Declaration of Helsinki and Japanese Ethical Guidelines for Medical and Health Research Involving Human Subjects, which waived the need to obtain informed consent from patients because only non-personally identifiable data from electronic patient records were used and no personal information will be disclosed.

### Medical safety system for in-hospital deterioration

Our hospital has adopted a two-tiered RRS to detect in-hospital deterioration; the “Code Blue Team” deals with serious deteriorating events that require immediate resuscitation, including cardiac and/or pulmonary arrest, whereas the RRS is intended to prevent such critical events. The RRS was initially started for only adult patients in 2012 and was expanded to cover all ages in 2017. The type of response team is the so-called “RRT”, led mainly by nurses, consisting of nurses and physicians of the ICU, critical care centre, or operating room. The RRT is activated when the calling criteria are met; the nurse-in-charge first evaluates the deteriorating patient and, after consulting with the physician in charge of the RRT, decides on the necessary treatment plan. Furthermore, the actual therapeutic intervention and the need for ICU admission will be decided after consultation with the attending physician team and the ICU physician. However, using the calling criteria alone, the RRS for patients treated by highly specialized departments, such as paediatric cardiac surgery and cardiology, was almost never activated. In 2017, there were only 21 annual activations based on the calling criteria for all paediatric patients. Furthermore, there were no calls in patients whose primary department was paediatric cardiac surgery. Therefore, in addition to the usual activation of the RRT described above, we initiated the RRT follow-up after ICU transfer for patients with CHD with the highest risk of in-hospital deterioration in April 2018. The patients were periodically examined for at least 48 h after ICU transfer; visits at least twice a day, additional visits if condition deteriorates. The decision for readmission to the ICU was made through a consensus of the RRT staff, intensivist, and attending physician. During the study period, no major medical management policy changes were implemented in our hospital.

### Selection of participants

We included consecutive patients who underwent congenital heart surgery, who were admitted to the PICU postoperatively, and who were discharged alive from the PICU between April 2015 and March 2020 and those for whom relevant data pertaining to the post-ICU transfer period of 30 days were available. Patients who met the selection criteria were stratified into the pre- and post-intervention groups.

### Data collection

Several types of data were collected from electronic patient records at initial ICU admission. Demographic data included those on age (months), sex (male/female), weight (kg), single ventricle anatomy (Yes/No), diagnosis of chromosomal abnormalities (Yes/No), a chronic illness other than CHD (Yes/No), surgical history of tracheostomy (Yes/No), surgical history of gastrostomy (Yes/No), and home oxygen therapy (Yes/No). Operative data included those pertaining to Risk Adjustment for Congenital Heart Surgery 1 (RACHS-1) score (6 levels: 1–6; the higher the level, the greater the surgical complexity and the higher the risk of postoperative mortality), operative time (min), cardiopulmonary bypass (CPB) time (min), aortic cross-clamping time (min), water balance (mL/kg), and volume of blood products administered (mL/kg). ICU management data included those on the requirement for intensive care treatment (nitric oxide inhalation [Yes/No], extracorporeal membrane oxygenation [Yes/No]), ventilator days, length of ICU stay (days), and the requirement for new tracheostomy (Yes/No).

In addition, data at ICU readmission were collected on organ-specific classification (circulatory [e.g., acute heart failure]/respiratory [e.g., asthma attack]/infection [e.g., mediastinitis]/neurological [e.g., convulsive seizure]/cardiopulmonary arrest), presence of surgical complications (Yes/No), and surgical complication classification (cardiac tamponade/mediastinitis/phrenic nerve paralysis/intracranial haemorrhage).

### Outcome measures

The primary evaluation item was unplanned ICU readmission within 30 days after ICU discharge. The secondary evaluation items were the severity at ICU readmission: predicted mortality (%, calculated from the Paediatric Index of Mortality 3 [PIM3]), the requirement for each intensive treatment (intubation/mechanical ventilation, nitric oxide inhalation, extracorporeal membrane oxygenation), and clinical outcomes at ICU readmission (ventilator days, length of ICU stay [days], and ICU mortality). As a secondary evaluation item, Unrecognized Situation Awareness Failure Events (UNSAFE) transfers (Yes/No) was also adopted; UNSAFE transfer is one of the leading indicators for the effectiveness of paediatric RRS. In children, cardiac arrest and death are extremely infrequent, making it difficult to show the effect of interventions, so UNSAFE was devised as a more frequently occurring indicator, defined as ward to ICU transfers after which patients are intubated, placed on vasopressors, or receive three or more fluid boluses in the first one hour after transfer^8^.

We used PIM3 to calculate the predicted mortality rate because it is a representative score for calculating predicted mortality in the paediatric intensive care field and is also used in the Japanese multicentre registry, in which our institution participates.

### Statistical analyses

Descriptive statistics were used to summarize the characteristics of the patients. Continuous variables are expressed as medians with interquartile ranges (IQRs), whereas categorical variables are expressed as frequencies and percentages. Between-group differences in these types of variables were compared using the Wilcoxon rank-sum test and Fisher’s exact test, respectively. Multivariate logistic regression analyses for confounders were performed to examine the independent effects of the intervention on the primary outcome and variables significantly different between the two groups, and the results are presented as odds ratios (ORs) and their associated 95% confidence intervals (CIs). Variables that were significantly different between the two groups were selected as the confounders. All *P* values were two-sided, and a value < 0.05 was considered statistically significant. Statistical analyses were performed using the R version 3.1.0 (R Foundation for Statistical Computing, Vienna, Austria; www.R-project.org).

## Results

**Figure 1:**
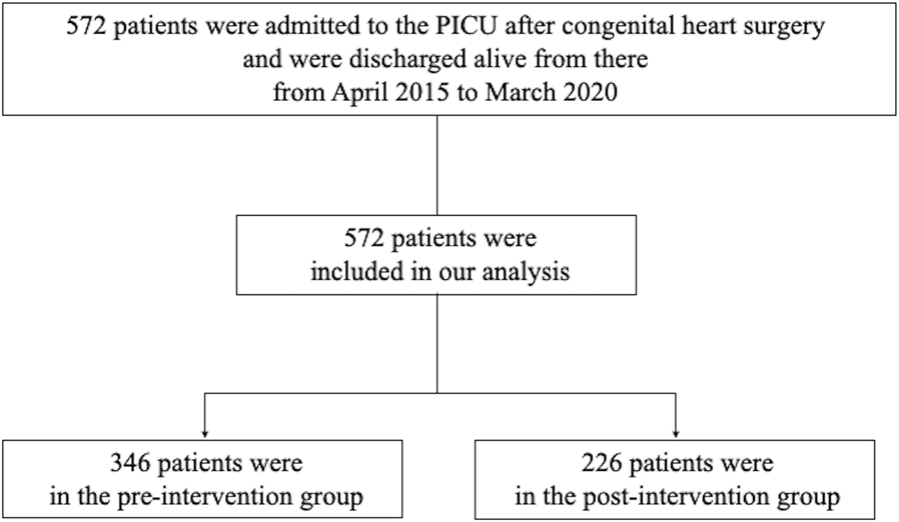
Study enrolment.

Overall, 572 patients were admitted to the PICU after congenital heart surgery and discharged alive from there during the study period, were included in the final analysis. We divided them into two groups: 346 patients were in the pre-intervention group, and 226 patients were in the post-intervention group (Fig. 1).

The patient demographics, operative variables, and ICU management variables are shown in Table 1. The patient demographics were similar between the two groups. The median age of the patients in both groups was approximately 12 months; the percentage of single-ventricular anatomy was approximately 10%. Few patients had a surgical history of tracheostomy or gastrostomy. Regarding operative variables, compared to the pre-intervention group, the post-intervention group had fewer cases with RACHS-1 score 1 and more with RACHS-1 score 3. Higher risk procedures tended to be performed more frequently in the post-intervention group than in the pre-intervention group. Longer operative and CPB time and larger volumes of blood products in the operation room were required for the post-intervention group than for the pre-intervention group. Regarding ICU management, more ventilator and PICU days were required in the post-intervention group than in the pre-intervention group.

**Table 1:** Initial patient demographics and operative and PICU patient management variables.

Variables	Pre-intervention (n = 346)	Post-intervention (n = 226)	*P* value
**Patient demographics**			
Male, n (%)	186 (53.8)	111 (49.1)	0.30
Age (month), median (IQR)	11 (4, 53)	13 (4, 69)	0.33
Weight (kg), median (IQR)	7.6 (4.8, 14.1)	8.1 (5.2, 17.8)	0.14
Single-ventricle anatomy, n (%)	35 (10.1)	25 (11.1)	0.78
Chromosomal anomaly, n (%)	64 (18.5)	35 (15.5)	0.36
Chronic illness other than CHD, n (%)	175 (50.6)	115 (50.9)	> 0.99
Surgical history of tracheostomy, n (%)	9 (2.6)	2 (0.9)	0.21
Surgical history of gastrostomy, n (%)	2 (0.6)	2 (0.9)	0.65
Home oxygen therapy, n (%)	34 (9.8)	17 (7.5)	0.37
**Operative variables**			
**RACHS-1 classification, n (%)**			
1	64 (18.9)	27 (11.9)	0.02
2	130 (38.5)	84 (37.2)	0.72
3	122 (36.1)	106 (46.9)	0.01
4	12 (3.6)	7 (3.1)	0.81
5	1 (0.3)	1 (0.4)	> 0.99
6	7 (2.1)	1 (0.4)	0.15
Operative time (min), median (IQR)	173 (130, 278)	232 (161, 326)	< 0.001
Cardiopulmonary bypass time (min), median (IQR)	64 (36, 133)	92 (48, 163)	0.001
Aortic cross-clamp time (min), median (IQR)	25 (0, 60)	31 (0, 77)	0.04
Volume of blood products administered (mL/kg), median (IQR)	16 (0, 35)	26 (1, 59)	< 0.001
**PICU management variables**			
Nitric oxide inhalation, n (%)	46 (13.3)	37 (16.4)	0.33
Extracorporeal membrane oxygenation, n (%)	7 (2.0)	5 (2.2)	> 0.99
Ventilator days (day), median (IQR)	2 (1, 4)	3 (2, 5)	< 0.001
Length of PICU stay (day), median (IQR)	4 (2, 7)	6 (4, 9)	< 0.001
New tracheostomy, n (%)	3 (0.9)	2 (0.9)	> 0.99

*IQR* interquartile range, *CHD* congenital heart disease, *RACHS-1* risk-adjusted congenital heart surgery-1, *PICU* paediatric intensive care unit.

### Primary outcome

Unplanned ICU readmissions within 30 days from ICU discharge were similar between the pre- and post-intervention groups (4.3% vs 6.2%, *P* = 0.33). The logistic regression analyses showed that, compared to the pre-intervention group, the crude and adjusted ORs for unplanned ICU readmission within 30 days among the post-intervention group were 1.5 (95% CI 0.7–3.1) and 1.2 (95% CI 0.5–2.7), respectively. Variables adjusted for in the logistic regression analyses were age (months), RACHS-1 score four or more (Yes/No), operative time (min), volume of administered blood products (mL/kg), and length of PICU stay (days).

### Reasons for paediatric intensive care unit readmission in both groups

Regarding the organ-specific classification of the ICU readmission cause, the post-intervention group tended to have more circulatory disorders (50.0% vs 13.3%, *P* = 0.05) and fewer respiratory disorders (21.4% vs 53.3%, *P* = 0.12) and cardiopulmonary arrests (0% vs 6.7%, *P* > 0.99). Moreover, surgical complications tended to be more common in the post-intervention group than in the pre-intervention group (85.7% vs 60.0%, *P* = 0.21); among them, cardiac tamponade was the most common (Table 2).

**Table 2:** Reasons for PICU readmission.

Reasons for PICU readmission	Pre-intervention (n = 15)	Post-intervention (n = 14)	*P* value
**Classification by affected organ, n (%)**			
Circulatory disorder	2 (13.3)	7 (50.0)	0.05
Respiratory disorder	8 (53.3)	3 (21.4)	0.12
Infection	2 (13.3)	3 (21.4)	0.65
Neurological disease	2 (13.3)	1 (7.1)	> 0.99
Cardiopulmonary arrest	1 (6.7)	0 (0)	> 0.99
Surgical complications, n (%)	9 (60.0)	12 (85.7)	0.21
Cardiac tamponade	2 (13.3)	5 (35.7)	0.21
Mediastinitis	2 (13.3)	3 (21.4)	0.65
Phrenic nerve paralysis	1 (6.7)	1 (7.1)	> 0.99
Intracranial haemorrhage	1 (6.7)	1 (7.1)	> 0.99

*PICU* paediatric intensive care unit.

### Secondary outcomes

Patients in the post-intervention group had a significantly lower predicted mortality rate (1.7% vs 5.3%, *P* = 0.001), lower UNSAFE transfer (86.7% vs 42.9%, *P* = 0.02) and required fewer ventilator days (median, 0.5 days [IQR 0.5, 4] vs median, 3 days [IQR 0, 1], *P* = 0.02) and a shorter length of ICU stay (median, 3 days [IQR 2, 4] vs median, 6 days [IQR 3, 9], *P* = 0.03) than those in the pre-intervention group. The ICU mortality rate was lower in the post-intervention group than in the pre-intervention group, although the difference was not statistically significant (0% vs 6.7%, *P* > 0.99). Furthermore, there was no significant difference in the treatments required between the two groups. All these results are shown in Table 3.

**Table 3:** Secondary outcomes: severity and outcomes at PICU readmission.

Outcomes	Pre-intervention (n = 15)	Post-intervention (n = 14)	*P* value
Predicted mortality rate (%), median (IQR)	5.3 (4.0, 8.4)	1.7 (0.7, 2.3)	0.001
UNSAFE transfer^a^, n (%)	13 (86.7)	6 (42.9)	0.02
**Treatments required in the PICU, n (%)**			
Intubation/mechanical ventilation	11 (73.3)	7 (50.0)	0.26
Nitric oxide inhalation	1 (6.7)	0 (0)	> 0.99
Extracorporeal membrane oxygenation	2 (13.3)	0 (0)	0.48
Ventilator days (day), median (IQR)	3 (0.5, 4)	0.5 (0, 1)	0.02
Length of PICU stay (day), median (IQR)	6 (3, 9)	3 (2, 4)	0.03
PICU mortality, n (%)	1 (6.7)	0 (0)	> 0.99

*IQR* interquartile range, *UNSAFE* unrecognized situation awareness failure events, *PICU* paediatric intensive care unit.

^a^UNSAFE transfer is defined as ward to ICU transfers after which patients are intubated, placed on vasopressors, or receive three or more fluid boluses in the first 1 h after transfer.

## Discussion

Our study demonstrates that 48-h follow-ups after ICU discharge by the RRT for postoperative patients with CHD did not reduce the unplanned ICU readmission rate within 30 days, but it decreased the predicted mortality, UNSAFE transfer rate, ventilator days, and length of ICU stay at ICU readmission.

This is the first report to show the effect of RRT on post-ICU follow-up after congenital heart surgery. In the context of the rapidly increasing number of patients with CHD, appropriate treatment of in-hospital sudden deteriorations in patients with CHD is an urgent issue. However, cardiac surgery and cardiology, which are highly specialized departments that mainly treat patients with CHD, tend to avoid intervention by third parties, including RRS. Therefore, active activation of the RRS by the staff of these departments is rare, making it difficult to make the RRS function effectively^9^. In contrary, if the follow-up after ICU discharge is predetermined by the RRT, the RRS can function effectively regardless of the wishes of this department. Several positive effects of the intervention in this study will lead to improvement in the quality of medical care for patients with CHD in-hospital emergencies in the future.

In contrast to our study, post-ICU follow-ups reduced ICU readmissions in a systematic review of adults^5^. There are some possible reasons for this. First, the RACHS-1 score, length of PICU stay, and duration of ventilation in the post-intervention group were higher than in the pre-intervention group, suggesting that they were at higher risk of deterioration and ICU readmission. Therefore, it may have been difficult to show improvement by this intervention. Second, it is possible that the patient evaluation by the RRT, a third party other than the attending physician, may have had prompted ICU admission before the patient became seriously ill. In fact, there was a decrease in the illness severity among patients who required unplanned ICU readmission after the intervention in the current study, suggesting overcoming resistance of the primary teams. Third, surgical complications as a reason for ICU readmission, especially cardiac tamponade, tended to be more common in the post-intervention group than in the pre-intervention group. Since our treatment policy is that postoperative cardiac tamponade is always treated in the operating room with tamponade release and drainage, following which the patients must be admitted to the ICU, RRT interventions cannot reduce ICU readmissions. Therefore, the high incidence of cardiac tamponade was considered a reason for the lack of decline in the ICU readmission rate. A previous study of paediatric patients who were readmitted to the cardiac care unit found that the percentage of patients with increased pericardial effusion was 6.4%, which was lower than that in our post-intervention group (35.7%). The cause of the high incidence of cardiac tamponade in this study is unknown; no major changes in the perioperative treatment strategy, including anticoagulation management, existed before or after the intervention. The rate of respiratory disorders as the reason for ICU readmission decreased in the post-intervention group, suggesting that the post-ICU follow-ups might have prevented the progression of respiratory disorders.

The intervention reduced the severity of the illness and improved some outcomes of unscheduled PICU readmissions. The reason for this, as mentioned above, could be the fact that the third-party’s assessment could have prompted an ICU admission at an earlier stage, before the condition became severe. A single-centre before-and-after study of adults found that RRT activities, including follow-up after ICU discharge, reduced the Acute Physiology And Chronic Health Evaluation II score, the need for mechanical ventilation, and CPA in the ICU at ICU readmission^10^, similar to our results.

### Study limitation

Several limitations of this study should be acknowledged. First, this was a single-centre study, and the results may not be generalizable to all centres because medical management varies between institutions. Since our hospital is a relatively small facility with 100–150 paediatric cardiac surgeries per year, it may be expected that a larger number of CHD patients will have more in-hospital sudden changes at a facility with a larger number of surgeries, and thus more benefit may be expected from RRT follow-up. Alternatively, large facilities with comprehensive care for CHD patients may have fewer in-hospital sudden changes to begin with. Differences depending on the size of the facility will be an important issue for further study. Second, the before-and-after study design is inherently unable to exclude potential bias (e.g. selection/assignment, history, Hawthorne, and reporting/publication bias)^11^. One report has pointed out the dangers of examining the effectiveness of paediatric RRS interventions using only a before-and-after study design^12^. The third limitation is the differences in the severity of illness (e.g. operative risk and PICU days) between the two groups. No major changes in treatment strategy or medical staffing were made during the study period, and the exact cause was unknown. Although we adjusted for these differences as confounding, we cannot deny the possibility that unmeasured and unknown confounding may have distorted the results. Fourth, the possibility of a measurement bias cannot be denied: the primary endpoints and the criteria for PICU readmission were not standardized.

A multifaceted approach is needed to improve the quality of care for the rapidly increasing number of patients with CHD. It is recommended to establish a sufficient number of specialized CHD centres, to establish a safe and smooth transport system^13–16^. Moreover, this post-ICU follow-up may be one of the effective countermeasures against in-hospital sudden deteriorations in patients with CHD. Further prospective, randomized, multi-centre studies are needed to demonstrate the real effects of the intervention.

In conclusion, this single-centre before-and-after study showed that post-ICU follow-ups of patients who underwent congenital heart surgery did not decrease their unplanned ICU readmission but improved the severity of the illness and several outcomes at ICU readmission. This may be one effective method to improve the prognosis of in-hospital deteriorating patients after congenital heart surgery.

## Supplementary Information

Supplementary Information.

## Data Availability

The authors confirm that the data supporting the findings of this study are available within the supplementary materials.
